# Frequency of Hyperuricemia and its Risk Factors in the Adult Population

**DOI:** 10.7759/cureus.4198

**Published:** 2019-03-06

**Authors:** Sooraj Raja, Akshay Kumar, Ramesh D Aahooja, Ujala Thakuria, Simran Ochani, Faizan Shaukat

**Affiliations:** 1 Internal Medicine, Jinnah Sindh Medical University, Karachi, PAK; 2 Internal Medicine, Shaheed Mohtarma Benazir Bhutto Medical College Lyari, Karachi, PAK; 3 Internal Medicine, Shaheed Mohtarma Benazir Bhutto Medical University, Larkana, PAK; 4 Internal Medicine, Jinnah Postgraduate Medical Center, Karachi, PAK

**Keywords:** hyperuricemia, uric acid, prevalence, risk factors, cardiovascular risk, pakistan

## Abstract

Introduction

Hyperuricemia (HU) does not only predispose to gouty arthritis but also increases the risk of major cardiovascular events and chronic kidney disease and decreases the overall quality of life. Although the incidence of hyperuricemia is increasing in the Asian population, prevalence studies from healthy asymptomatic adults are still scarce. The aim of this study was to determine the frequency of HU in the general population of Karachi, Pakistan.

Methods

A cross-sectional study was conducted in various out-patient clinics across Karachi in January 2019. Serum uric acid (SUA) levels were recorded using UASure Blood Uric Acid Monitoring System. Age, gender, body mass index (BMI), the frequency of activity, the frequency of meat consumption, and the presence of hypertension were noted. For hypertensives, the use of thiazide diuretics was noted. Data were entered and analyzed using SPSS v. 22.

Results

This study was completed by 375 individuals including 208 (55.5%) men and 167 (44.5%) women. Their mean age was 48.78 ± 13.399 years and the mean SUA level was 5.92 ± 1.73 mg/dl. There were 83 (39.9%) men and 30 (17.9%) women with elevated SUA levels. The overall prevalence of HU was 30.1%. Patient characteristics including gender, hypertension, the use of diuretics frequency of meat consumption, sedentary lifestyle, and BMI showed statistical significance with mean SUA levels. Eta-squared indicated a weak effect of SUA with gender, age, and use of thiazide diuretics. A moderate effect was seen with hypertension, the frequency of meat consumption, sedentary lifestyle, and BMI.

Conclusion

Hyperuricemia is a health hazard and its incidence is high in Pakistan. Patients who have risk factors for elevated SUA levels must be monitored for hyperuricemia at regular intervals.

## Introduction

The association between elevated serum uric levels (SUA) and cardiovascular diseases (CVD) has long been documented in the epidemiological database [1-2]. With the passage of time, short- and long-term studies have been conducted to strengthen this association. Many such studies have concluded hyperuricemia (HU) to be an independent risk factor for CVD including major cardiovascular events (MACE) such as stroke and ischemic cardiac diseases [3-4]. However, whether or not elevated SUA levels substantially increase the risk of CVD is an unresolved dispute among the researchers. Some studies, which concluded the association between elevated SUA and incidence of CVD, had other essential risk factors such as hyperlipidemia, metabolic syndrome, and hypertension [5].

Furthermore, chronic kidney disease has also been proven to have an independent association with the increase in uric acid levels [6]. Insulin and HU are postulated to share a bidirectional causal effect relation, and hence type II diabetes is also associated with HU [7]. Furthermore, obesity and dyslipidemia that are considered salient components of metabolic syndrome share association with the uric acid level [8]. Hypertension, diuretic (thiazide) use, and obesity have been linked with predisposing to increased uric acid levels and also the development of gout [9-11]. Male gender has shown a stronger association with uric acid level compared to that of female, especially in the middle age [12].

Even after understanding the delirious effects of SUA levels other than gouty arthritis, measuring SUA is not a routine general practice unless vague and chronic musculoskeletal pains are complained or evident gout flare presents. Hence, HU may go undiagnosed. Undiagnosed and asymptomatic HU pose health risks including resistant hypertension and chronic kidney disease [13]. According to a systemic review published in 2015, there is published population-based evidence of HU prevalence from 24 countries. A higher prevalence of HU in Asia, especially East Asia, has been reported; however, there was no data from South Asia [14]. A Bangladeshi study in 2018 estimated 9.3% of the population to be hyperuricemic [15]. In India, the prevalence of HU estimated in 2018 was 25.8%, with an increased prevalence in diabetics, hypertensives, and diabetic hypertensives [16]. This is still no large-scale, nationwide study conducted in Pakistan. In order to contribute to the literature, this study is an attempt to assess the frequency of HU in the largest city of Southern Pakistan, Karachi.

## Materials and methods

A cross-sectional study was conducted in various out-patient clinics across Karachi in January 2019. A total of 375 adults participated after informed consent. Their age was 18 years or more.

Uric acid levels were recorded using the UASure Blood Uric Acid Monitoring System from a capillary blood sample via fingertip puncture. This method has been studied to be reasonably comparable to the laboratory- based assays [17]. HU was defined as SUA levels greater than 5.8 mg/dl in women and 7 mg/dl in men [18]. Age, gender, body weight, height, the frequency of activity, and the presence of hypertension were noted in all participants. For hypertensives, the use of thiazide diuretics was noted. Participants who were physically inactive or active for less than two days per week were regarded as sedentary. BMI was calculated from body weight and height. Individuals with a BMI of 30 or more were regarded as obese. The frequency of meat consumption was noted on a seven-point frequency scale that ranged from (1) “never”, (2) “less than once a month”, (3) “1-3 times per month”, (4) “once a week”, (5) “several times per week”, (6) “once and a day”, and (7) “several times per day”. 

Data were entered and analyzed using SPSS v. 22. Gender-wise frequency of HU was calculated and correlated with its risk factors. Mean and standard deviation (SD) were calculated and compared using independent *t*-test. *P* value ≤0.05 was taken as significant. The effect size was calculated using Eta-squared. Eta-squared is the effect size measurement for analysis of variance (ANOVA). It determines the strength of an effect on a continuous variable. It is interpreted as the proportion of variance in the effect of target variable while controlling the effect of other variables. Eta-squared ≤0.04 signifies a statistically significant but weak effect; 0.05-0.36 signifies a moderate effect and Eta-squared >0.36 indicates a strong effect [19].

## Results

This study was completed by 375 individuals. There were 208 (55.5%) men and 167 (44.5%) women participants. Their mean age was 48.78 ± 13.399 years (range: 20-72 years). There were 106 (28.3%) participants of age less than 40 years and 269 (71.7%) of age more than 40 years. The mean SUA level of the sample was 5.92 ± 1.73 mg/dL (range: 3.00-10.70). The mean SUA level in men was 6.01 ± 2.93 mg/dL (range: 4.26-10.70) and that in women was 4.99 ± 2.17 mg/dL (range: 3.00-8.58). There were 83 out of 208 men (39.9%) who had elevated SUA levels. In total, 30 out of 167 women (17.9%) had elevated SUA levels. Overall, there were 262 (69.9%) normouricemic and 113 (30.1%) hyperuricemic participants. These two groups were then categorized according to their genders, age, and presence of HTN, use of thiazide diuretics, lifestyle, and BMI, as shown in Table 1.

**Table 1:** Correlation of serum uric acid levels with individual risk factors (N = 375)

	Gender n (%)		Age in years n (%)		Hypertension n (%)		Thiazide diuretics n (%)		Sedentary lifestyle n (%)		BMI in m/kg^2^ n (%)	
	Male	Female	< 40	> 40	Yes	No	Yes	No	Yes	No	≤30	≥30
Normouricemia	125 (47.7%)	137 (52.3%)	77 (29.4%)	185 (70.6%)	89 (34.0%)	173 (66.0%)	52 (19.8%)	210 (80.2%)	116 (44.3%)	146 (55.7%)	104 (39.7%)	158 (60.3%)
Hyperuricemia	83 (73.5%)	30 (26.5%)	29 (25.7%)	84 (74.3%)	76 (67.3%)	37 (32.7%)	23 (20.4%)	90 (79.6%)	52 (46.0%)	61 (54.0%)	59 (52.2%)	54 (47.8%)

As seen in Table 1, in the hyperuricemic group, there were more males than females (73.5% vs 26.5%); more hyperuricemics were found in older age individuals (74.3% vs. 25.7%), more hypertensives than normotensives (67.3% vs. 32.7%). Only 20.4% of hyperuricemic participants were using thiazide diuretics; sedentary lifestyle was less common in hyperuricemic participants (46.0% vs. 54.0), and more hyperuricemics were of BMI less than 30 (52.2% vs. 47.8%).

The correlation of SUA levels with the frequency of meat consumption is shown in Table 2.

**Table 2:** Correlation of serum uric acid levels with the frequency of meat consumption (N = 375)

Serum uric acid levels (mg/dL)	Frequency of meat consumption n (%)					
	Never	Less than once a month	1-3 times a month	Once a week	2-3 times a week	Once a day
Normouricemia	14 (5.3%)	61 (23.3%)	46 (17.6%)	59 (22.5%)	74 (28.2%)	8 (3.1%)
Hyperuricemia	8 (7.1%)	0 (0.0%)	7 (6.2%)	21 (18.6%)	46 (40.7%)	31 (27.4%)

All patient characteristics including gender, age, hypertension, the use of diuretics, and frequency of meat consumption, sedentary lifestyle, and BMI were in correlation with the mean SUA levels. Statistical significance via *p*-value showed that all factors were significantly correlated except for age. Eta-squared indicated a weak effect of SUA levels with gender, age, and use of thiazide diuretics. A moderate effect was seen with hypertension, the frequency of meat consumption, sedentary lifestyle, and BMI (Table 3).

**Table 3:** Correlation of patient characteristics with mean serum uric acid levels (mg/dl; N = 375) *p value ≤0.05 is significant; **Eta-squared ≤0.04 weak effect, 0.05–0.36 moderate effect, >0.36 strong effect

Patient characteristics	Serum uric acid (Mean ± SD)	Number (n)	P value*	Partial Eta-squared**
Gender				
Male	6.19 ± 1.84	208	0.001	0.031
Female	5.57 ± 1.55	167		
Hypertension				
Yes	6.64 ±1.63	165	0.000	0.137
No	5.35 ± 1.60	210		
Age in years				
Less than 40	5.86 ± 1.79	106	0.673	0.000
More than 40	5.94 ± 1.71	269		
Frequency of meat consumption				
Never	5.66 ± 1.08	22	0.000	0.212
Less than once a month	4.71 ± 1.10	61		
1-3 times a month	5.13 ± 1.69	53		
Once a week	6.19 ± 1.93	80		
2-3 times a week	6.21 ± 1.61	120		
Once a day	7.53 ± 0.97	39		
Thiazide diuretics				
Yes	5.52 ± 1.79	75	0.027	0.013
No	6.02 ± 1.17	300		
Sedentary lifestyle				
Yes	6.13 ± 1.83	168	0.029	0.13
No	5.74 ± 1.63	207		
BMI in meters/kg^2^				
Less than 30	6.54 ± 1.62	163	0.000	0.100
More than 30	5.43 ± 1.67	212		

## Discussion

The study reveals that almost 40% of men and 20% of women are hyperuricemic, without any clinical sign or symptom. Most of the hyperuricemic participants were of older age group, hypertensive, not using thiazide diuretics, not leading a sedentary lifestyle and with BMI ≤30 mg/kg^2^. Hyperuricemia was twice more common in men than women.

The mean SUA level in this study is 5.92 ± 1.73 mg/dL. In a nationwide study from Pakistan published in 2017, the mean uric acid levels were reported to be 6.11 ± 1.7 mg/dL. The overall frequency of hyperuricemia was 39% in their study with 27.9% male hyperuricemics and 49.3% women [20]. The overall frequency in this study is comparable (30.1%); however, the gender-wise distribution is the opposite in this study with more hyperuricemic men than women. Hyperuricemia has a varied prevalence in other Asian countries; China has a prevalence of 6% to 25%, Taiwan 10% to 52%, and Indonesia 18% [14]. The prevalence of hyperuricemia in India was 44.6% according to the study published in 2012 [21], which was reduced to 25.8% in 2018 [16].

A population-based study from China reported a statistically significant correlation of hyperuricemia with waist circumference, blood pressure, and triglyceride in men and with waist circumference, triglyceride and fasting plasma glucose in women. Hyperuricemia was also more common in men in their study. Individuals with this condition were 1.6 times more prone to developing metabolic syndrome [8]. Similarly, in a Bangladeshi study, the positive association of hyperuricemia with obesity has been established [15]. In a meta-analysis conducted with 18 prospective cohort studies, the adjusted risk ratio (RR) of hypertension was 1.41 in people with hyperuricemia. It was concluded that for every 1 mg/dl increase in the SUA level, RR for the incidence of hypertension was 1.13 [22]. The relationship of thiazide and loop diuretics has been established with acute gouty arthritis. There was a longitudinal change in the SUA level of 0.72 mg/dl in patients who were initiated with diuretic therapy for their HTN compared to patients who were not. Patients taking any other antihypertensive drug only had a 0.64 times risk of gout [23]. Longitudinal trials have shown a significant improvement in the overall health-related quality of life in hyperuricemic patients when treated with urate-lowering therapy for up to two years [24]. Furthermore, the management of hyperuricemia with purine-like xanthine oxidase inhibitors has also significantly reduced the risk of major cardiovascular events, especially hypertension [25].

The overall prevalence of hyperuricemia is comparable to regional prevalence. However, as compared to Qudwai et al., our study showed a decreasing trend in prevalence [20]. This might be attributed to the smaller sample size and data collection from one city only. This study has important limitations. However, the study did manage to conclude the essential factors, contributing to hyperuricemia. Further studies are recommended in this area to endorse the relationship of hyperuricemia with hypertension, the use of thiazide diuretics, obesity, and lifestyle.

## Conclusions

In conclusion, hyperuricemia is a health hazard that may go undiagnosed. Keeping in view its delirious complications, patients who have risk factors for elevated SUA levels must be monitored for hyperuricemia at regular intervals. Timely diagnosis and initiation with urate-lowering therapy will help delay complications, protect against cardiovascular events, and enhance the quality of life.
